# Goal planning in mental health service delivery: A systematic integrative review

**DOI:** 10.3389/fpsyt.2022.1057915

**Published:** 2022-12-19

**Authors:** Victoria Stewart, Sara S. McMillan, Jie Hu, Ricki Ng, Sarira El-Den, Claire O’Reilly, Amanda J. Wheeler

**Affiliations:** ^1^School of Pharmacy and Medical Sciences, Griffith University, Gold Coast, QLD, Australia; ^2^Centre for Mental Health, Griffith University, Brisbane, QLD, Australia; ^3^Menzies Health Institute Queensland, Griffith University, Brisbane, QLD, Australia; ^4^The University of Sydney School of Pharmacy, Faculty of Medicine and Health, The University of Sydney, Camperdown, NSW, Australia; ^5^Faculty of Medical and Health Sciences, The University of Auckland, Auckland, New Zealand

**Keywords:** goal planning, goal setting, mental health, mental illness, recovery planning, rehabilitation, systematic integrative review

## Abstract

**Introduction:**

Goal planning is routinely employed in mental health service delivery to identify priorities for treatment and support the achievement and evaluation of outcomes. Previous systematic reviews of the literature have focused on the use of goal planning in a range of physical and cognitive disability settings, but there is a lack of information regarding how goal planning is used in mental healthcare.

**Aims:**

This systematic integrative review aimed to understand the types of goals, effectiveness of goal planning, the experience of goal planning and barriers and facilitators to effective goal planning in mental healthcare settings.

**Methods:**

Five databases were systematically searched using key terms related to mental health AND goal planning. The search was supplemented through citation chaining. Due to the heterogeneity of the studies, a narrative synthesis approach to data analysis was undertaken.

**Results:**

Fifty-four studies were identified through the search of the literature following the PRISMA guidelines. Data was systematically extracted and thematically organized. There was a high level of heterogeneity among the studies, originating from a range of countries and with diverse characteristics and focus. Four themes emerged from the data analysis and included: (i) goal planning as a central aspect of interventions; (ii) types of goals planned; (iii) factors that influenced goal planning and/or attainment; and (iv) collaboration and concordance in goal planning.

**Conclusion:**

This review found some support for the use of goal planning to improve outcomes in mental healthcare although there was no identified standardized approach to the use of goal planning. Individualized, recovery-oriented and collaborative goal planning was recommended but not always used in practice. Further research to understanding the most appropriate skills and training needed to support collaborative and effective goal planning is needed.

**Systematic review registration:**

[https://www.crd.york.ac.uk/prospero/], identifier [CRD42020220595].

## Key messages

- This systematic review identified themes regarding the use of goal planning in mental healthcare, increasing our understanding of how goal planning is used in service delivery.
- Four themes emerged from the data and included (i) goal planning as a central aspect of interventions; (ii) types of goals planned; (iii) factors that influenced goal planning and/or attainment; and (iv) collaboration and concordance in goal planning.
- Overall, findings suggest that there are benefits associated with the use of goal planning in mental healthcare settings, and goals are important in supporting change and achieving outcomes.
- Goals prioritized by practitioners and service users were not always well-aligned and shared decision making and collaborative approaches improved engagement, satisfaction, and outcomes for service users.
- Goal planning is a complex process and a better understanding of the skills required by practitioners to support recovery-oriented goal planning is needed.

## 1 Introduction

Goal planning is widely recognized as an integral component of psychiatric rehabilitation (1), with evidence suggesting that goal planning facilitates behavioral change (2). It is seen as a process of discussion and negotiation through which service users and health practitioners identify priorities for treatment to support the achievement of desired future states (3). A range of terminology in relation to goal planning has been used in the literature and includes goal setting and care planning, along with action planning, in which plans are made to operationalize and assist achievement of goals (4). Given the close relationship between the process of goal setting and action planning, these terms are often used interchangeably. Within mental healthcare, terms such as recovery planning and shared decision making are common. Goal planning has been shown to enhance service user motivation, adherence, self-efficacy, and health related quality of life (5).

Goal planning processes support individuals to identify desired outcomes, specific behaviors to change and how to go about making changes (6) and should be intentionally developed in negotiation with those who will be directly impacted (3). Goal planning theory suggests that goals that are conscious and specific as well as sufficiently difficult produce better results (7, 8), and can be influenced by factors such as intrinsic motivation and self-efficacy (7).

Current mental healthcare is focused on promoting recovery, utilizing interventions that focus on increasing competencies or skills and providing environmental supports to assist service users achieve a meaningful life (9). Shared decision making is seen as an essential process in supporting personal recovery and self-determination, occurring when all participants are informed, involved, and influential in the decision making and goal planning process (10). Whilst shared decision making is promoted in mental healthcare, several barriers have been identified including concerns regarding service user capacity, complicated by power imbalances due to statutory provisions for involuntary treatment in many countries (11). Previous literature reviews regarding the use of goal planning in healthcare have focused on the rehabilitation context for acquired disability (5), acquired brain injury (12, 13), spinal cord injury (14), and older patients (15). Given the theoretical and practical differences in service delivery for those with physical injuries as compared with people experiencing mental illnesses, it is important to understand any differences in goal planning processes and outcomes.

The involvement of people with mental illness in goal planning is generally seen as beneficial, however information regarding what makes goal planning effective is lacking. The purpose of this review was to systematically examine and synthesize the literature regarding goal planning in mental healthcare.

### 1.1 Objectives

The purpose of this systematic integrative review was to locate, access, compile, and map the published studies that exist about the use of goal planning within the context of mental health service delivery. This review aimed to understand (i) the types of goals being developed; (ii) the effectiveness of goal planning in improving mental health outcomes; (iii) how service users and practitioners experience goal planning processes, and (iv) the barriers and facilitators to effective goal planning in mental health service delivery from the perspectives of service users and practitioners.

For the purpose of this review, the definition of a rehabilitation goal by Siegert and Levack was applied in that goals are “actively selected, intentionally created, have purpose and are shared (where possible) by the people participating in the activities and interventions” (3).

## 2 Materials and methods

This integrative review process was developed in accordance with the Preferred Reporting Items for Systematic Review and Meta-Analysis (PRISMA) statement (16) and was registered with PROSPERO (CRD42020220595). The PRISMA checklist is attached (Supplementary material).

### 2.1 Search strategy

Six electronic databases [Medline (Ebsco), CINAHL, Embase, PsycINFO (Ovid), and Scopus] were searched in March 2022. The PICO framework (17) structured the search strategy with the population (“P”) identified as adults experiencing mental illness, the intervention (“I”) defined as goal planning, the comparison group (“C”) was not relevant for this study, and outcomes (“O”) were not limited. The search strategies were developed with assistance from an academic librarian and combined MESH terms and keywords related to mental health (e.g., mental illness, mental health condition, and psychiatric diagnosis) AND goal planning (e.g., goal setting, care planning, and shared decision-making). Additional studies were identified through reviewing the reference lists and citations of retrieved articles. Further details regarding the search strategy is provided (Supplementary material). The main inclusion criterion was that the study included adults diagnosed with a mental illness or practitioners working with this population and reported on the impact or experience of goal planning. Empirical studies also needed to be published in a peer-reviewed journal in English, with no restriction on the study methodology used. Conceptual, commentary and review papers were excluded, as were studies focused on specific cohorts such as forensic, substance use or dementia populations. Further details regarding the inclusion and exclusion criteria can be found in the published protocol (18).

### 2.2 Study selection

**FIGURE 1:**
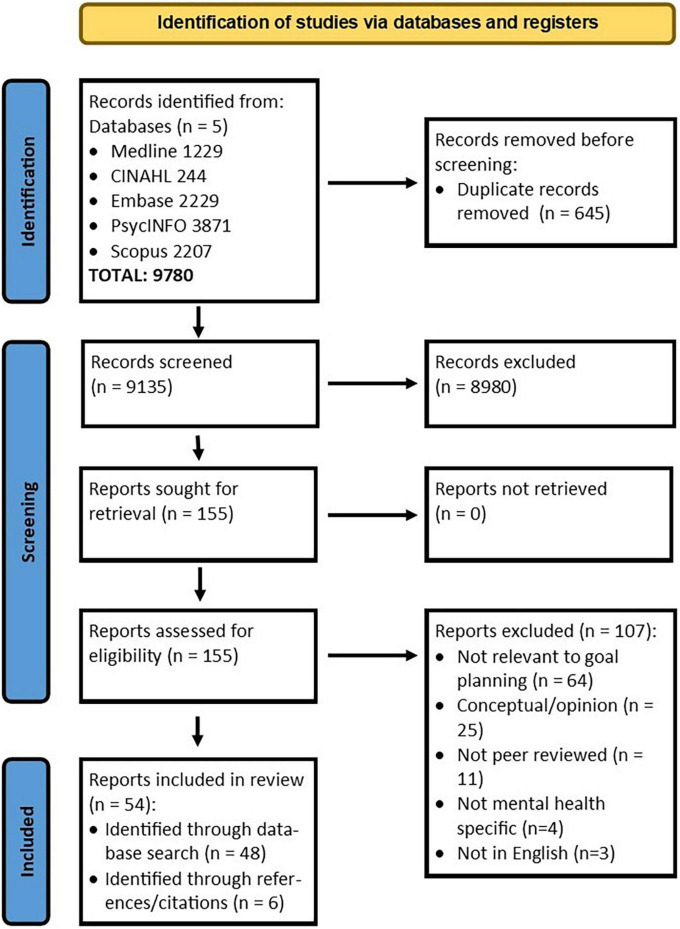
PRISMA flow diagram.

First, one author (VS) conducted a preliminary screening of titles to exclude any irrelevant studies (e.g., focused on different populations) or duplications. Articles were then imported into the Covidence review management tool (19) with titles and abstracts independently screened for relevance by two authors (VS and RN). An over-inclusive approach was utilized with full-text articles obtained for any abstracts in doubt. The same authors then screened all full-text articles for inclusion in the final review. Consensus on inclusion was reached by discussion between the authors, with a third author (SM) arbitrating on those in doubt (*n* = 3). Further details for study exclusion can be found in Figure 1.

### 2.3 Critical appraisal

The quality of the included studies was assessed using the Mixed-Methods Appraisal Tool (MMAT), which was designed to appraise the methodological quality of five different study categories (qualitative research, randomized controlled trials, non-randomized studies, quantitative descriptive studies, and mixed methods studies) (20). The MMAT scale comprises two screening questions, followed by individual items for the five different methodologies. Initially, three authors (VS, JH, and SM) independently reviewed a selection of 20 studies, with a consensus on scoring reached through discussion. Another author (AW) arbitrated disagreements (*n* = 2). Two authors (VS and JH) then independently completed the appraisal of the final 34 studies, again reaching a consensus on any disagreements (*n* = 6). The methodological quality of included studies ranged from 20 to 100% (*M* = 66.3%). For qualitative studies, the methodological quality ranged from 60 to 100% (*M* = 92.7%), randomized controlled trials ranged from 20 to 100% (*M* = 64.4%), and quantitative studies ranged from 40 to 80% (*M* = 60.0%). Following MMAT author guidelines, the overall quality score for mixed methods studies was identified as the lowest score achieved (*M* = 56%, range 20–80) across the study components (20). As integrative reviews consider a wide range of evidence, studies were not excluded on the appraised level of quality. However, consideration of study quality was taken into account during the interpretation of the data. A summary of the quality of studies is presented (Supplementary material) and should be considered alongside the findings of this review.

### 2.4 Data extraction and analysis

This process aimed to analyze and synthesize the information from included studies to develop new knowledge and insights about mental healthcare goal planning, which were not visible through reading each study in isolation. An integrative literature review method was undertaken, following the process described by Whittemore and Knafl (21). An integrative approach was selected as it allows for the inclusion of studies from diverse methodologies and captures the complexity of varied perspectives (21, 22).

Initially, an Excel spreadsheet was used by one author (VS) to extract descriptive data on the author, publication date, country, study design, aims, study setting, sample characteristics, goal planning method, healthcare practitioner, description of intervention(s), goal planning outcome measure(s), service user, and practitioner results (18). A second author (SM) completed a quality check (20% of included studies) of data extraction. Next, an in-depth analysis of the eligible studies was undertaken by one author (VS) in consultation with another author (SM). Due to the heterogeneity of the studies included in this review, a narrative synthesis approach (23) was identified as an appropriate approach to understanding and presenting the findings of the included studies. First, data were extracted according to the review questions (e.g., use of goal planning and experiences of goal planning) and presented in tabular form. These results were then reviewed by the same author (VS) and manually added to tables that structured the study themes based on the aims and outcomes described within each study. The included studies were summarized within this thematic framework by one author (VS).

## 3 Results

### 3.1 Study characteristics

Fifty-four studies were identified (Figure 1) and published between 1982 and 2021. Studies showed heterogeneity in characteristics and outcomes (Tables 1–4). The majority of studies were quantitative (*n* = 29), 16 utilized mixed methods and nine involved a qualitative study design. Studies were undertaken in the USA (*n* = 17), Europe (*n* = 12) Australia (*n* = 9), UK (*n* = 9), Israel (*n* = 3), Brazil (*n* = 2), Canada (*n* = 1), and India (*n* = 1). Recruitment occurred in a range of settings including outpatient settings (e.g., primary health; *n* = 23), specialized psychiatric rehabilitation programs (*n* = 16), inpatient settings (*n* = 6), residential settings (*n* = 3), recovery college (*n* = 1), and recovery camp (*n* = 1), with four studies recruiting from general community forums. Studies reported on participants diagnosed with schizophrenia/psychotic disorder (*n* = 10), depression (*n* = 9), borderline personality disorder (*n* = 1), bipolar disorder (*n* = 1), and post-traumatic stress disorder (*n* = 1). The remaining 32 studies reported on groups of participants diagnosed with a range of mental illnesses (*n* = 15) or what the authors described as severe and persistent mental illness (*n* = 17). Forty-eight of the studies included health practitioners from backgrounds described as multidisciplinary teams (*n* = 21), psychotherapists (*n* = 5), psychologists (*n* = 5), case managers or care coordinators (*n* = 4), occupational therapists (*n* = 2), peer workers (*n* = 2), student (*n* = 1), or undefined backgrounds (e.g., mental health worker; *n* = 8).

**TABLE 1:** Goal planning as a central aspect of interventions.

Intervention	Intervention description and time-frame	Study type	Study aim	Sample characteristics	Outcome measurements	Key outcomes	References; country
Intensive Psychiatric Rehabilitation (IPR)	IPR consisted of four components: assessing and developing readiness for rehabilitation, goal setting, goal achievement, and goalkeeping	Pre-post design	Measure outcomes of residential and employment goals over 18 months	People diagnosed with mental illness receiving mental health services (*n* = 238)	The IAPSRS toolkit captured role functioning across life domains	Participants with residential goals had significant positive changes in residential outcomes (mean change score 1.12, SD = 1.32, *p* < 0.001) and employment outcomes (mean change score 0.56, SD = 1.96, *p* < 0.0157). Participants with employment goals had significant positive changes in employment status (mean change score 1.39, SD = 2.10, *p* < 0044) and gross earnings (mean change score 146.75, SD = 333, *p* < 0.0417).	Anthony et al. (31); USA
Psychiatric Rehabilitation (PR)	PR approach assists consumers to identify goals, skills and resources needed, plan and carry out interventions Sessions offered every three weeks, no maximum	RCT	To determine if PR assists people to obtain their personal rehabilitation goals	People with SPMI attending rehabilitation services (in- and out-patients); IG (*n* = 80) vs. CG (*n* = 76)	Consumer interviews using service provider questionnaires including: exploration of goal attainment; Personal Empowerment Scale, CAN, WHOQoL-BREF, Social Functioning Scale	Goal attainment numerically higher in PR participants particularly societal participation (work) and social contacts at 12 months (ARD 16.4%, 95% CI 1.9–30.8%) and 24 months (ARD 20.7%, 95% CI 3.8–37.6%). No notable difference in secondary outcomes except for societal participation (greater effect in the intervention group).	Swildens et al. (32); Netherlands
Methodical work approach	Systematic and transparent goal-driven way of working with cyclic evaluation and adjustment. Five phases including translation of problems into goals, search for means to realize goals, formulation, implementation and evaluation of individualized plans	Quasi-experimental, non-equivalent control-group design	Examine the impact of the methodical work approach in reducing episodes of seclusion	Inpatient wards in psychiatric hospital; intervention ward (*n* = 134); comparison wards (*n* = 544).	Incidences of, and number of hours of, seclusion over time	Compared to control wards, methodical work approach significantly reduced the number of incidents (*B* = −0.55, SE = 0.20, *p* < 0.01) and total hours of seclusion (*B* = −63.46, SE = 17.25, *p* < 0.01).	Boumans et al. (30); Netherlands
Mindfulness-based cognitive therapy (MBCT)	MBCT combines cognitive behavioral techniques with mindfulness strategies to understand and manage thoughts and emotions Eight (2-h) weekly classes and 6 h of meditation practice/week.	RCT	Impact of MBCT on goal specificity and whether goals were perceived as more achievable	Community members that met diagnostic criteria for major depression; IG (*n* = 14) vs. waitlist (*n* = 13)	BDI-II, Measure to Elicit Positive Future Goals and Plans (including a rating of specificity), AMT	Increased specificity of goals (mean difference from baseline to follow-up 1.17, *p* < 0.01) and increased perceived likelihood of achieving goals (mean difference −5.71, *p* < 0.01) post MBCT, with no significant change (mean difference = 0.001, *p* = 1.0 and 1.31, *p* > 0.4, respectively) in the waitlist group.	Crane et al. (34); UK
Goal setting And Planning (GAP)	Brief intervention that targets goal-setting and planning skills to improve well-being	Cross over trial	Determine the impact of GAP in improving well-being	People attending mental health services (*n* = 82)	Positive and Negative Affect Scale, Satisfaction with Life Scale, BHS, measure of outcome expectancy and efficacy for goals	GAP participants had significantly positive changes for satisfaction (*p* < 0.01), outcome expectancy and efficacy for goals (*p* < 0.01), positive affect (*p* < 0.05), and hopelessness (*p* < 0.05).	Farquharson and MacLeod (29); UK
Mental contrasting with implementation intentions (MCII)	Self-regulation strategy that combines mental contrasting and forming implementation intentions to foster goal commitment and striving	RCT	Whether MCII assists people with depression to attain their goals and manage mood	Community members with acute mild to moderate depression; MCII (*n* = 28) vs. waitlist (*n* = 19)	Self-reported goal attainment, BDI-II (German), Generalized Self-Efficacy Scale (German)	MCII participants reached their goals more often (78.6 vs. 31.6%), OR 7.94 (95% CI 2.23–32.3, *p* < 0.01) but no significant difference in depression between groups.	Fritzsche et al. (40); Germany
Individual Resiliency Training (IRT)	IRT is a psychosocial treatment for individuals following a first episode of psychosis, supporting motivation through recovery goal setting and pursuit	Clinical trial	Evaluate whether the recommended number of IRT sessions improved motivation and psychosocial outcomes	People with first episode psychosis accessing IRT (*n* = 310) vs. community care (*n* = 180)	Structured clinical interview, QLS, Brief Assessment of Cognition in Schizophrenia	Exposure to four or more goal focused IRT sessions was associated with significant improvements in motivation (*B* = −0.111, SE = 0.047, *p* < 0.018) and role functioning (*B* = −0.187, SE = 0.084, *p* < 0.026) compared to community care.	Fulford et al. (28); USA
Strengths-based Case Management (SBCM)	SBCM emphasizes an individual’s strengths and resources to promote active engagement in defining and attaining goals supported by recovery-relevant community services	RCT	To assess the impact of SBCM on client outcomes, goal planning and attainment, quality of life, relationships, unmet needs, self-efficacy, symptoms, and service use	Participants with SPMI receiving rehabilitation services; SBCM (*n* = 696) vs. treatment as usual (*n* = 580)	MANSA, self-rating of quality and type of interpersonal relationships, unmet needs, self-efficacy, goal planning, and attainment, CSI	SBCM participants had significantly improved self-efficacy [3.07 (SD = 0.49) vs. 2.97 (SD = 0.52), *p* < 0.001], unmet needs [2.83 (SD = 0.54) vs. 2.81 (SD = 0.57), *p* < 0.05], and general quality of life [2.89 (SD = 0.52) vs. 2.86 (SD = 0.56), *p* < 0.01[, and set more goals than the control group. SBCM participants also consumed fewer services at follow-up.	Gelkopf et al. (27); Israel
Individual Plan (IP)	A statutory tool to ensure that service users participate in planning their own treatment and care, have a written plan and an appointed service provider to coordinate care	Longitudinal qualitative study – interviews	Describe what service users see as important and explore the process of IP development and use	People diagnosed with SPMI (*n* = 10), their relatives, coordinators, or therapists (*n* = 12	Coding of interviews using Malterud’s Systematic Text Condensation	Formulating (realistic) goals and then actioning goals were essential elements of a well-functioning IP. Collaboration and ongoing evaluation were important as well as providing sufficient information and negotiating responsibility.	Holum (26); Norway
Organizational Behavior Management (OBM)	Therapists provided with training in setting measurable goals, providing verbal and written feedback and praise.	Multiple baseline design	To determine if OBM techniques (goal setting, feedback, and reinforcement) improve therapist performance, and patient outcomes	Psychotherapists (*n* = 4) employed in privately managed supported homes and their patients (*n* = 37)	For staff – quality of treatment plans, session notes, time to submit, JSI; for patients – goal progress, PAC, medication compliance, incident reports, hospitalizations	OBM processes (monitoring paperwork and goal setting processes) resulted in large increases in the number of goals addressed weekly, quality of treatment plan reviews and decreased paperwork submission times resulting in significant increase in patient goal progress (*F* = 12.21, *p* < 0.01) and activity levels (*F* = 3.71, *p* < 0.05).	Huberman and O’Brien (43); USA
GOALS	Modules and manual focused on personalized goals using elements of motivational interviewing and cognitive-behavioral strategies	Pilot study – uncontrolled trial	To determine if GOALS intervention improves goal regulation and prevents symptoms of mania	Community members (*n* = 10) diagnosed with bipolar disorder	BRMS, MHRSD, BDI-SF, ASRM, WASSUP, and Consumer Satisfaction Questionnaire	Mean levels of manic symptoms decreased significantly (*p* < 0.05, *d* = 0.88). Additionally, all participants found the program highly relevant and helpful.	Johnson and Fulford (36); USA
		Feasibility RCT	Determine feasibility of GOALS intervention	People diagnosed with schizophrenia/psychosis with anxiety, and/or depression; IG (*n* = 37) or treatment as usual (*n* = 38)	Satisfaction, time budget measure, choice of outcome in CBT in psychosis, HADS, MI, PANSS, PSYRATS, CORE-10, WEMWBS, MANSA, and SOFAS	Brief training effective in skilling workers to deliver intervention. 74% of IG participants partially or fully achieved their goals and significant improvements in goal attainment noted with a moderate effect size of 0.56 at 18 weeks.	Waller et al. (35); UK
Valutazione delle Abilità e Definizione degli Obiettivi (VADO) in English: Skills Assessment and Definition of Goals	Rehabilitation approach focusing on negotiating realistic specific goals and routinely evaluate attainment of these goals derived from the Boston University principles	RCT	To assess if a VADO aids recovery	People diagnosed with SPMI recruited from rehabilitation centers; IG (*n* = 57) vs. CG(*n* = 41)	VADO goals, modified SOFAS, BPRS	Intervention group showed greater improvements in psychopathology (mean BPRS global score 57.8 ± 21.2 at baseline vs. 49.8 ± 20.7 at 12 months) and social functioning (mean FPS score 41.1 ± 12.8 at baseline vs. 49.0 ± 14.3 at 12 months).	Pioli et al. (33); Italy
Occupational Goal Intervention (OGI)	Rehabilitation program based on Goal Management Training that uses learning strategies to perform activities and everyday tasks	RCT	Whether OGI improves executive functions and functional performance	People diagnosed with treatment resistant schizophrenia; IG (*n* = 14) vs. CG (*n* = 11)	BADS, ILSS (Brazil), DAFS-BR (Brazil), WAIS	OGI participants had significantly higher results in executive function with medium-to-large effect sizes (*d* = 0.72–0.87) and limited improvements in functional levels after 15 weeks (*d* = 0.54–0.85).	Vizzotto et al. (37); Brazil
		RCT		People diagnosed with TR schizophrenia; IG (*n* = 28) vs. CG (*n* = 26)	BADS, ILSS (Brazil), DAFS-BR (Brazil), WAIS	OGI participants showed improvements on almost all aspects of EF and communication skills, finance management, and grooming. All were maintained at 6-month follow-up, during which there was no intervention.	Vizzotto et al. (38); Brazil
Goal-Focused Supportive Contact (GFSC) and Cognitive Behavioral Social Skills Training (CBSST)	Both interventions used semi-structured group sessions based on Goal Attainment Scaling (GAS) processes	Longitudinal clinical trial	Measure goal progress of those attending group psychosocial interventions	People diagnosed with schizophrenia or schizoaffective disorder (*n* = 55)	PANSS, GAS	Goals included relationships, self-care, employment, leisure, housing, school, transportation, addictions, and money management. A significant increase in goal attainment scores noted (*d* = 1.42, *p* < 0.001).	Tabak et al. (41); USA
Spirituality group intervention	Weekly spirituality group exploring spiritual definitions and concepts and relationship with recovery	Experimental design	Impact of spiritual intervention on goal attainment	People attending a psychosocial rehabilitation program; IG (*n* = 20) vs. CG (*n* = 28)	Self-identified goals and attainment	IG participants had higher goal attainment (100 vs. 57% in CG).	Wong-McDonald (39); USA
Collaborative Recovery Model (CRM)	CRM promotes goal setting through supporting recovery-oriented and collaborative approaches	File audit	Assess the quality of goal setting and determine if staff training improves goal quality	People diagnosed with a psychotic disorder with more than 5 unmet needs (CAN) (*n* = 122)	Goal Instrument for Quality (Goal-IQ)	Significant increase in the number of goals and quality of goals post-CRM training. 70% of goals demonstrated collaboration.	Clarke et al. (42); Australia
Whole Health Coaching	Whole Health Coaching focuses on eight areas of self-care and guides people through identifying, setting and monitoring goals	Single-case experimental design	Impact of peer whole health coaching on goal outcome	Veterans diagnosed with PTSD accessing primary health care clinics (*n* = 10)	PTSD Checklist, GAS	Whole Health Coaching associated with increased (*t* = 2.49, *p* < 0.05) and sustained goal attainment (approximately 4 months).	Johnson et al. (44); USA
Illness Management and Recovery (IMR) program	IMR is a curriculum-based rehabilitation program in which participants learn about illness management through setting and pursuing personal goals	Qualitative	To describe participants’ lived experience of personal goal setting	People diagnosed with SPMI (*n* = 15)	Collaborative goal planning, IMR goal tracking sheet	IMR helped participants break down their personal goals into manageable short-term goals and were “guided to set clearer and specific goals,” “encouraged to pursue personal goals,” and “were supported to resume when they stopped making progress.”	Jensen et al. (25); Denmark

AMT, Autobiographical Memory Test; ARD, adjusted risk difference; ASRM, Altman Self-Rating Mania Scale; BADS, Behavioral Assessment of the Dysexecutive Syndrome; BDI, Beck Depression Inventory; BHS, Beck Hopelessness Scale; BPRS, Brief Psychiatric Rating Scale; BRMS, Bech-Rafaelson Mania Scale; CAN, Camberwell Assessment of Need Short Appraisal Schedule; CORE-10, Clinical Outcomes in Routine Evaluation outcomes Measure; CSI, Colorado Symptom Index; DAFS, Direct Assessment of Functional Status; GAS, Goal Attainment Scaling; HADS, Hospital Anxiety and Depression Scale; ILSS, Independent Living Skills Survey; JSI, Job Satisfaction Index; MANSA, Manchester Short Assessment of Quality of Life Scale; MHRSD, Modified Hamilton Rating Scale for Depression; MI, Mobility Inventory; PAC, Patient Activity Checklist; PANSS, Positive and Negative Syndromes Scale; PSYRATS, Psychotic Symptoms Rating Scales; QLS, Quality of Life Scale; SOFAS, Social and Occupational Functioning Assessment Scale; WEMWBS, Warwick-Edinburgh Mental Well-being Scale; WAIS, Wechsler Abbreviated Intelligence Scale; WASSUP, Willingly Approached Set of Statistically Unlikely Pursuits; WHOQOL-BREF, World Health Organization Quality of Life Instrument.

**TABLE 2:** Types of goals planned.

References; country	Sample characteristics	Study aim	Study type and analysis	Goal planning method	Key findings
Battle et al. (52); USA	People (*n* = 26) diagnosed with depression attending an outpatient Group, Individual, Family Treatment of Depression (GIFT) psychotherapy program	Understand the types of treatment goals developed by people experiencing depression	Qualitative – written goals coded and categorized using an “editing organizing style”	Therapist and client worked together to set long-term, realistic goals (4 in total)	Goals included improving social (73% of participants) and family (65%) relationships, improving physical health (50%), finding a job (35%), and organizing one’s home (35%)
Brophy et al. (51); Australia	People with psychosocial disability aged 26–63 accessing funding through the National Disability Insurance Scheme	Understand goals identified as important by service users	Mixed method – semi-structured interviews (*n* = 41) descriptive statistics presented; 15 of these analyzed further using qualitative methodology	Interview asking what would make a good life and rating goal importance	Health, economic, social connection, housing, and personal relationship goals identified as important
Deegan et al. (50); USA	Random selection of power statements (*n* = 272) from people diagnosed with serious mental illness accessing community mental health services	Understand service users’ goals for medication treatment	Qualitative – retrospective review of written goals contained in power statements	Power statements	Medication goals were to control symptoms (56%), improve functioning (54%), and lowering/stopping medication doses (31%). Reasons for medication goals included improving or maintaining social relationships (51%), wellbeing (32%), self-sufficiency (23%), employment (19%), and engaging in hobbies (15%).
Holtforth et al. (55); Switzerland	Inpatient (*n* = 675) treatment goals in a private psychiatric hospital	Determine if the Bern Inventory of Treatment Goals (BIT-T) is useful in understanding the goals of inpatients	Mixed method – written treatment goals coded to BIT-T	Self-identified three areas of change on worksheet, discussion with therapist	The majority of goals coded to BIT-T concerned symptom relief (77.3%), interpersonal goals (36%), personal growth (26.1%), wellness (18.8%), and existential issues (6.2%).
Iyer et al. (49); India	Individuals (*n* = 68) accessing a first-episode psychosis clinic	Identify and describe treatment goals, how individuals evaluate the importance, and level of achievement	Mixed method – written goals thematically categorized. Self-rated importance and achievement explored statistically.	Self-reported goals, importance, and achievement	Goals were related to employment (38.2%), close family and interpersonal relationships (20.6%), school (16.2%), and symptom relief and psychological recovery (10.3%). Goals were perceived as being very important (8.46–10/10).
Macpherson (48); UK	All people (*n* = 136) attending a psychiatric rehabilitation program	Describe the types and outcomes of rehabilitation goals after one year	Mixed method, coding of goals and descriptive statistics	Goals developed in care plan review between worker and participant	Goals related to a range of activities including social functioning and daily activities (24%), change in medication (13%), and medical/physical problems (7%). A total of 68% of goals were fully achieved after 12 months, 11% partially achieved, and 20% not achieved.
McNaughton et al. (57); USA	People diagnosed with depression (*n* = 7) interviewed and survey disseminated through PatientsLikeMe online network (*n* = 200)	Explore treatment goal setting experiences and types of goals that were important	Mixed method – online survey and semi-structured interviews. Descriptive and content analysis of data	Self-reported goals on survey	A total of 42% reported treatment goals in the areas of physical health (62.7%), cognitive functioning (60.2%), and social aspects of life (57.8%). A total of 61% believed the goal attainment approach would be helpful to set and evaluate treatment goals.
Moran et al. (56); Israel	People diagnosed with a mental illness (*n* = 2121) living in supported housing	Examine types of goals/plans and impact on recovery	Cross sectional mixed method. Descriptive and content analysis of data.	Self-reported during interview	A total of 80% supported housing and 72% group home residents had goals for the coming year. Goals related to advancing forward in life (21%), improving social life (18%), and learning or training (14%). Those with goals showed higher levels of personal recovery (*p* < 0.0001), and staff assistance supported personal recovery for both those in supported housing (*b* = 0.13, SE = 0.03) and group homes (*b* = 0.06, SE = 0.03)
Moxham et al. (47); Australia	People diagnosed with SPMI attending Recovery Camp (*n* = 27)	Examine the types of goals set and extent they were attained while at Recovery Camp	Mixed method study. Descriptive and content analysis of data	Self-reported goals and attainment level	The most common theme related to connecting with others and the majority of these goals were either entirely or almost entirely attained. Other goal themes included challenging myself, developing healthy habits and recovery.
Ng et al. (46); Australia	People diagnosed with borderline personality disorder attending a community-based psychotherapy program (*n* = 102)	Categorize personally meaningful treatment goals	Qualitative. Descriptive and content analysis of data.	Self-reported goals guided by Target Complaints Method	Reduction of symptoms was the most commonly reported goal by participants (86.3%), followed by the desire to improve well-being (62.7%), having better interpersonal relationships (52.9%), and having a greater sense of self (39.2%).
Ramsay et al. (54); USA	People experiencing first-episode psychosis (*n* = 100) recruited through hospitals and crisis centers	Understand the life and treatment goals and perceptions of how mental health professionals could assist them	Qualitative. Inductive content analysis of data.	Self-reported during interview	Employment, education, relationships, housing, health, and transportation were the most frequently stated life goals. Treatment goals included medication management, reducing symptoms, a desire to be well, and physical health. Most wanted vocational and educational services, assistance with symptom management, and drug abuse.
Sommer et al. (45); Australia	People attending a Recovery College mental health service (*n* = 64)	Examine the types of goals, degree achieved, what goals more likely to be achieved and effect of goal difficulty	Mixed method. Content analysis and logit regression analysis.	Student learning plans co-produced with peer workers using GAS framework	Education (19.4%), physical health (18.2%), social (18.2%), and mental health goals (17.8%) were most common. Probability of 0.73 that goals fully or partially achieved with those rated as less difficult more likely to be achieved.
Uebelacker et al. (53); USA	People with a primary diagnosis of depression attending private psychiatric hospital (*n* = 50)	Develop a list of frequently reported treatment goals and understand relative importance	Qualitative. Content analysis.	Self-reported during interview	On average participants identified 8.7 goals. Goals included improving relationships with others (83%), decreasing anxiety (54%), decreasing sadness (46%), and finding or being able to return to a job (41%). Suicidal thoughts, self-understanding, and relationships with family goals were most frequently ranked among the top three issues for treatment to address.

**TABLE 3:** Factors influencing goal planning and/or attainment.

References; country	Sample characteristics	Aim of study	Study description	Goal planning method	Outcome measures	Findings
Clarke et al. (65); Australia	Participants (*n* = 71) with a diagnosis of a psychotic disorder and receiving case management support from a mental health provider	To examine whether (i) symptoms and functioning impact on goal progress; and (ii) goal attainment improves mental health	Non-randomized pre-post (quantitative)	Collaborative goal technology (CGT)	CGI, HoNOS, LSP, RAS, Kessler 10	Average goal attainment of 48.18 (SD = 29.52). Higher levels of self-perceived symptom distress impeded goal attainment (*r* = −0.14, *p* < 0.01); greater goal attainment led to improved self-confidence and hope (β = −0.41, *p* = 0.00), and predicted recovery outcome (β = 0.26, *p* = 0.04).
Dickson et al. (60); UK	Participants (*n* = 23) with depression recruited from mental health services, and control participants (*n* = 26) recruited from the community	Whether depressed persons differ from never depressed persons on the number of freely generated approach and avoidance goals, appraisal of these goals, and reasons why these goals would and would not be achieved	Prospective non-randomized (quantitative)	Self-generation of approach and avoidance goals and reasons why goals would or would not be achieved	Goal Task, Goal Explanation Task, Goal Ratings, BDI	No significant differences in number of goals or perceived importance of goals. Depressed participants judged their approach goals as less likely to occur (*F* = 4.16, *p* = 0.04) and gave lower ratings of their control over goals outcomes (*F* = 16.29, *p* < 0.001).
Dickson and Moberly (59); UK	Participants (*n* = 21) with depression recruited from mental health services and control participants (*n* = 24) recruited from the community				Goal Task, Goal Explanation Task	Those with depression had similar number and importance of goals but depressed participants reported fewer specific goals (*F* = 10.74, *p* = 0.002), less specific explanations for approach goal attainment and non-attainment.
Dickson et al. (61); UK	Participants (*n* = 42) with depression recruited from clinics and control participants (*n* = 51) recruited from same region			Self-generation of approach and avoidance goals and reasons why goals would or would not be achieved and expected outcome	Goal task, Goal Importance, Goal Expectancy, GAS, Written Fluency Task, PHQ-9	Compared to controls, depressed participants reported fewer approach goals (*F* = 16.43, *p* = 0.001) (but not more avoidance goals), rated their approach goal (rewarding) outcomes as less likely to happen (*F* = 10.09, *p* = 0.002)and avoidance goal (threatening) outcomes as more likely to happen (*F* = 29.69, *p* = 0.001).
Gard et al. (63); USA	Participants (*n* = 47) with schizophrenia or schizoaffective disorder attending outpatient clinics and control participants (*n* = 41) recruited from the community	To explore if people with schizophrenia demonstrate less anticipatory pleasure goals and pursue goals that are less effortful	Mixed method using Ecological Momentary Assessment	Semi-structured interview asking participant’s goals planned for the next few hours	Semi-structured interviews, EASy (modified), MATRICS Consensus Cognitive Battery, QLS (abbreviated)	In comparison to controls, participants reported setting less effortful goals (*t* = −3.38, *p* = 0.001) but set goals that were more pleasure based (*t* = −7.65, *p* = 0.001).
McGuire et al. (70); USA	Veterans with SPMI (*n* = 21) accessing a psychosocial rehabilitation and recovery center	To explore the impact of self-experience on conceptualizations of treatment goal setting	Qualitative	Semi structured interviews focused on goals for treatment	Goals analyzed according to dialogical self-experience type	Themes indicate people with differing self-experiences vary in how they form goals and the barriers they face in this process. Recognizing differences among the four self-types can individualize the approach to collaborative goal setting.
Pueschel et al. (64); Germany	People diagnosed with mental illness (*n* = 61) attending an outpatient psychotherapy center	Understand the association between goal progress and depressivity for motive-congruent goals	Quantitative descriptive study	Personal striving framework – all explicit goals were written down	Goal rating, self-rated goal progress, BDI, NEO-FFI	Persons who made more progress at goals that matched their implicit motives experienced fewer depressive symptoms.
Rose and Smith (66); Australia	People receiving mental health support from a community organization (*n* = 704)	Examine the relationship between goal setting, achievement, working alliance, and recovery	Quantitative non-randomized	Goal setting card	Goal data, WAI, RAS (Domains and Stages)	Both goal achievement and the strength of the working alliance had a positive effect on personal recovery. Goal achievement was related to a strong working alliance.
Sanches et al. (67); Netherlands	People diagnosed with SPMI and receiving rehabilitation services both inpatient and outpatient (*n* = 156)	Investigate whether working alliance predicts goal attainment and if goal attainment is related to QoL	Secondary analysis of RCT data	Boston University approach to Psychiatric Rehabilitation	WAI (practitioner perspective), Goal attainment, WHOQOL-BREF, Brief Psychiatric Rating Scale	Working alliance (goal component rather than personal bond) predicted goal attainment (*B* = 0.12, SE = 0.06, *p* = 0.04) and goal attainment led to improved QoL scores at 24 months (*B* = 7.63, SE = 3.15, *p* = 0.02).
Wollburg and Braukhaus (62); Germany	Inpatients (*n* = 657) diagnosed with depression receiving CBT	Investigate the influence of goal definition on treatment outcome	Quantitative non-randomized	Self-identified goals	BDI (German), Goal attainment	Framing goals using avoidance terms did not affect goal attainment but was associated with less symptomatic improvement.

BDI, Beck Depression Inventory; CGI, Collaborative Goal Index; EASy, Environmental Assessment Scale; GAS, Goal Adjustment Scale; HoNOS, Health of the Nation Outcome Scale; K10, Kessler 10; LSP-16, abbreviated Life Skills Profile; NEO-FFI, NEO Five-factor Inventory; PHQ-9, Personal Health Questionnaire; QLS, Quality of Life Scale, RAS, Recovery Assessment Scale; WAI, Working Alliance Inventory; WHOQOL-BREF, World Health Organization Quality of Life Instrument.

**TABLE 4:** Collaboration and concordance in goal planning.

References; country	Sample characteristics	Aim of study	Study description	Goal planning method	Outcome measures	Findings
Arns and Linney (75); USA	Individuals diagnosed with a SPMI (*n* = 141) attending psychosocial rehabilitation programs	Measure individualization of goals and determine the relationship with service user characteristics, staff, and program differences	Retrospective analysis of written goals (*n* = 364)	Individual treatment plans	Measure of goal individualization	The mean goal individualization scores was 2.58/5 with only 7.8% of goals including a measurable criterion and 12.8% including specific details.
Fakhoury et al. (73); UK	People residing in supported housing and diagnosed with schizophrenia or psychotic disorder (*n* = 41) and housing service staff (*n* = 39)	To explore the agreement between service users and staff on set goals.	Qualitative – interviews	Self-reported goals	Content analysis of self-reported goals	Poor or no agreement on client reported goals and staff (care coordinators and housing care managers) perceptions of needs/goals.
Falloon and Talbot (74); USA	People diagnosed with SPMI attending a day treatment program (*n* = 82)	To evaluate the effectiveness of a day treatment program in meeting the needs of service users	Mixed method	Collaborative goal achievement plan (five steps)	Concordance estimated by therapist, goal achievement plan, goal achievement rated	Strong relationship between client involvement and goal achievement – 59% of collaborative goals had good achievement compared with 6% of therapist dominated goals.
Knaeps et al. (68); Belgium	Inpatients in a psychiatric hospital (*n* = 733) and their clinicians (*n* = 279)	To compare patient and practitioner perceptions of realistic vocational goals, barriers and support needed	Descriptive quantitative	Self-identified goals	Questionnaires paired for patient and practitioner	A total of 45% of patients have competitive job goals while only 32% of practitioners find this realistic. Patients perceive fewer vocational barriers than clinicians and prefer less intense vocational support options.
Kuhnigk et al. (76); Germany	Physicians with psychiatric expertise (*n* = 160) randomly recruited through registry; individual physicians (*n* = 63) recruited patients (*n* = 105); in addition relatives (*n* = 50) and payers’ representatives (*n* = 30) recruited by research team	To investigate and compare the valuation and perceived attainment of treatment goals by various stakeholder groups (consumer, physicians, payers, and relatives)	Mixed method – combined and integrated approach	A list of 20 common treatment goals developed from a focus group. Participants were asked to rank these in accordance with importance.	Focus group and stakeholder interviews	All goals were considered very relevant by all stakeholder groups, but no goal was ranked in the top three by all stakeholders. “Improved cognitive abilities” was ranked highly by patients, physicians, and relatives while payers gave the highest priority to cost-related goals (i.e., work, hospitalizations).
Lecomte et al. (72); Canada	People (*n* = 165) attending a psychiatric rehabilitation program	Describe rehabilitation goals and assess if services were helping participants meet those goals	Mixed method	CASIG-SR used to assess function in a range of domains and elicit goals	Concordance measured by patient self-rating of importance and support received to achieve goals	The most frequently mentioned goals pertained to improving consumers’ financial situation, physical health, cognitive capacities, and symptoms. Among these goals, the level of concordance was highest for services addressing symptoms and lowest for religious or spiritual goals.
Lenehan et al. (71); Australia	Random selection of individuals (*n* = 66) receiving services from a non-government organization	Examine if homework tasks are coherent with the goals of individuals receiving mental health recovery support	Mixed method	My action plan (MAP)	CAN-GAP criteria used to determine if homework tasks match goals	Significantly more homework matches than mismatches at categorical level (*p* < 0.001) and domain level (*p* < 0.001).
McGuire et al. (70); USA	Veterans with SPMI (*n* = 21) accessing a psychosocial rehabilitation and recovery center	To understand the consumer perspective regarding treatment plan goals and how this relates to personal and clinical context	Qualitative	Treatment plan goals	Semi-structured interviews	Complex relationship between consumers, providers, and the treatment plan. A range of experiences were articulated including agreement, conflicting understandings, wording and prioritizing goals, and total rejection.
Proctor and Hargate (24); UK	People diagnosed with mental illness (*n* = 477) attending community health services	Investigate correlations in goal data	Mixed method using retrospective goal data	Goal Attainment Form (GAF)	GAF, Clinical Outcomes for Routine Evaluation Outcome Measure, therapist assessment	Patient descriptions of their problems and benefits of therapy were different from clinicians’ perspectives.
Shadmi et al. (69); Israel	People accessing psychiatric rehabilitation services from 2013 to 2015 (*n* = 2,345/3,236 survey respondents had matched provider responses)	Examine consumer and provider goal concordance and relationship to attainment	Cross-sectional quantitative	Self-rated goals and provider perspective of goals; self-rated attainment	Linked consumer and provider goals rated for concordance	Overall consumer-provider concordance was 54% – highest for employment (76%), housing (71%), and intimate relationship (52%) and lowest for family relationships (23%), and finances (15%). Consumer-provider concordance was associated with goal attainment (*p* < 0.001).
Wyder et al. (77); Australia	Inpatients in an acute adult psychiatric ward (*n* = 55)	Impact of Motivational Aftercare Planning (MAP) intervention on content of recovery plans	Pre-post design	Recovery plans	Content and language of plans pre- and post-intervention	Content of recovery plans shifted focus following MAP, changing from third to first person language, e.g., from decreasing symptoms, clinical stability risk management, and treatment compliance to general wellness and physical/emotional safety, participation in meaningful activities, social connections, and listening to their concerns.

### 3.2Goal planning themes

After analyzing the extracted data, four themes emerged and included: (i) goal planning as a central aspect of interventions; (ii) types of goals planned; (iii) factors that influenced goal planning and/or attainment; and (iv) collaboration and concordance in goal planning. As many studies reported information that could have fit into more than one theme category, the study’s primary focus was used in determining the most appropriate discrete theme category. Whilst it is acknowledged that this is not an ideal process, it was identified as the best way to synthesize a diverse range of results in a brief and manageable form and provide meaningful insights on how goal planning could be used in mental health service delivery. For example, Proctor et al. (24) utilized a range of data collected in a community mental health setting to explore the content of practitioner-developed and service user goals, goal achievement, benefits of therapy, and satisfaction with service delivery. When discussing the implications for practice, the results particularly highlighted the lack of agreement between practitioner and service user goals and the need for services to routinely collect this information to inform improvements. As such, this study was coded to the theme of exploring concordance in goal planning.

#### 3.2.1 Goal planning as a central aspect of interventions

Eighteen rehabilitation and therapy interventions in 20 studies were described in the literature (Table 1). The interventions were based on individual goal planning approaches (25–30), psychiatric rehabilitation principles (31–33), cognitive-behavioral techniques (34–36), learning strategies (37, 38), spirituality (39), self-regulation (40), Goal Attainment Scaling (GAS) (41), recovery (42), Organizational Behavior Management (OBM) (43), and coaching (44). Fourteen of the interventions reported on goal attainment, which was assessed using a variety of methods, including a range of clinical and functional scales (27, 29, 31, 33, 35–38), GAS (41, 44), self-reporting (32, 40, 43), or an unreported measure (39). Other studies reported on the experiences of goal planning (25, 26), collaboration (42), and outcomes, including specificity of goals (34), motivation levels (36), and episodes of seclusion (30). Interventions were reported to support goal achievement for participants’ general well-being and quality of life (27, 35, 39, 40, 44) and in a range of areas, including housing and employment (31, 32, 39, 41), social participation (32, 33, 39–41), symptom management (29, 33, 35–38, 40), and executive functioning (37, 38). No instances of interventions having a negative effect on goal achievement, i.e., worsening of service user outcome measures, were reported.

Due to the variability in study designs and outcome measures, direct comparisons of results were not possible. In terms of outcomes, studies that reported on the process of goal planning identified that formulating and actioning specific goals, along with collaboration, ongoing evaluation of goal progress, and the provision of sufficient information to enable informed choice in regards to treatment or intervention options was determined by participants as necessary in supporting goal achievement (26, 30, 34). One quality RCT identified that goal-based interventions improved attainment of work and social goals (32), another with a large sample size but poor quality found improved self-efficacy and quality of life, with fewer unmet needs (27), whilst another reported greater improvements in psychopathology (33). Participants reported goal planning as a process or structure encouraging change by identifying specific steps to enable achievement (28). Good quality qualitative studies identified that involvement in goal planning resulted in improved motivation and increased goal-directed behaviors (27) and decreased hopelessness (25), while a well-powered clinical trial demonstrated enhanced confidence in achievement (28). Additionally, a small RCT reported that structured goal planning interventions improved rehabilitation practitioners’ confidence and motivation to support service users (33). Focusing on positive outcomes rather than deficits or obstacles was also an essential element of interventions (29, 40).

Additionally, training staff in goal planning interventions increased the number and quality of goals generated by promoting participant and practitioner collaboration in the goal planning process (31, 35, 42, 43). Studies also identified that interventions that promoted an individualized approach to goal planning (27, 41, 43, 44), spanning a range of life domains (31, 32, 39), were preferred by participants.

Whilst goal planning interventions were identified as effective in promoting positive outcomes for those experiencing mental illness, it was recommended that further research to determine which aspects of the process were most effective in supporting positive results is needed (37, 38).

#### 3.2.2 Types of goals planned

Thirteen studies primarily described the goals identified and prioritized by people experiencing mental illness (Table 2). Goals were developed in community-based treatment and rehabilitation programs (*n* = 8) (45–52), inpatient (*n* = 3) (53–55), supported housing (*n* = 1) (56) settings and with people living in the community (*n* = 1) (57). Data were collected through interviews (*n* = 6) (46, 51–54, 56), surveys (*n* = 1) (55, 57), treatment notes (*n* = 1) (48), worksheets (*n* = 1) (47), and assessment scales (*n* = 2) (45, 49). One study used power statements, an approach assisting clients prepare goal statements regarding medication treatment prior to medication visits (50). Studies used qualitative (*n* = 5) (46, 50, 52–54) or mixed methods (descriptive statistics and content analysis) (*n* = 7) (45, 47–49, 51, 56, 57) approaches to categorize, code, or describe the types of goals identified by participants. One study (55) utilized the Bern Inventory of Treatment Goals (BIT-T), a previously published goal taxonomy developed for psychotherapy goals (58).

Overall, participants experiencing a range of mental illnesses identified a broad range of goals from different life domains, including, but not limited to, employment/education, housing, relationships, mental health, physical health, symptom relief, medication use, and living skills. Researchers recognized that the goals identified by participants were largely developmentally appropriate, realistic, and did not stem from psychotic symptoms (49, 54). Generally, life and personal recovery goals were more prevalent and prioritized by service users, with symptom control seen as a means to achieving life goals (45, 50, 53, 55).

Given the diverse range of goals identified by those experiencing mental illness, some qualitative study authors raised concerns regarding the required scope of expertise or skills practitioners would need to support service users to achieve their prioritized goals (51, 52, 54). A high quality qualitative study recommended that practitioners focus on assessing whether the service user experienced difficulty with motivation to make changes or whether additional skills and knowledge were required to assist them in achieving their goals (52). As such, communicating and coordinating care with other professionals was deemed necessary, which was reinforced by study participants reporting a preference for support from practitioners with diverse skills (51, 52). In addition to partnering with other practitioners to address goals, working in partnership with service users (51) and ensuring treatments were appropriate to support goal achievement were identified as important practitioner skills by service users (49). In line with recommendations from studies included in the above theme, structured goal planning processes were seen as necessary; fostering collaboration and shared decision making, promoting understanding, engagement and retention with services, improving adherence to treatments, increasing satisfaction, and enhancing outcomes (49, 53, 57).

#### 3.2.3 Factors influencing goal planning and/or attainment

Ten studies investigated aspects of the goal planning process that may influence the type of goals planned or outcomes achieved (Table 3). Studies explored factors such as whether the goals used avoidance or approach to formulation (59–62), how effortful goals were (63), whether goals were pleasure-based (63), as well as participant factors such as an individual’s dialogical self-experience (1), severity of symptoms (64, 65), and the quality of a participant’s working alliance with the practitioner (66, 67). Participants diagnosed with depression (1, 59–62), schizophrenia (63), or a range of mental illnesses (1, 64, 65, 67) were included and recruited from community-based services (59–61, 63–67) and/or hospitals (62, 67).

Service users who reported fewer symptoms or distress obtained greater goal progress, particularly if the goals were congruent with personal motives (64, 65). One moderately powered study (61) identified that a person’s self-experience or self-narrative impacted goal formation and the barriers faced in goal planning processes, recommending that practitioners use approaches appropriate for different levels of self-experience. Goal achievement was also related to a strong working alliance between service users and practitioners, suggesting greater collaboration and agreement in goal planning resulted in an improved quality of life and positive personal recovery for participants (66, 67).

Five studies explored the goal planning of people diagnosed with depression (59–62, 64). These studies reported that generally, participants identified more approach (moving to a desirable state) than avoidance (moving away from undesirable end state) goals, with no differences in types of goals found between those diagnosed with depression and control groups (59–61). A large study identified that framing goals as approach or avoidance did not influence goal attainment, although less symptomatic improvement was seen in service users identifying avoidance goals (62). Whilst a smaller study reported that participants diagnosed with depression rated approach goals as less likely to be achieved, undesirable goal outcomes more likely to occur, and perceived less control over their goal outcomes (60). Additionally, participants diagnosed with depression generated fewer specific goals and reasons for and against goal attainment (59), with a greater inclination to disengage with goals they viewed as unattainable (61). These outcomes suggest that people experiencing depression have a more pessimistic view of goal planning. As such, other authors recommended that practitioners focus on challenging negative thinking to support goal attainment (46) and promote goal specificity and perseverance to sustain motivation to participate in goal activities for this specific population (59, 61).

A medium sized study investigated the goal planning behaviors of participants diagnosed with schizophrenia (63). These service users planned less effortful goals and activities and engaged in goals and activities that were more pleasure-based. As a result, the authors recommended that practitioners support service users to break down more extensive and complex goals into smaller, lower-effort steps and engage in pleasurable activities or rewards to promote goal attainment (63).

#### 3.2.4 Collaboration and concordance in goal planning

Eleven studies evaluated collaboration and concordance between participant and practitioner perspectives regarding goal planning (Table 4). Seven of these studies were focused on the agreement between participant-identified goals, and the treatment plan developed or services provided (24, 68–73), whilst one study reviewed the level of collaboration in goal planning (74), one assessed the individualization of written goals (75), one examined and compared the goal priorities of participants, families, and practitioners (76), and one reviewed the effectiveness of a collaborative training intervention for practitioners (77). Goal planning processes in rehabilitation programs (69, 70, 72, 74, 75), community-based mental health services (24, 71, 76), supported housing (73), and hospitals (68, 77) were identified through existing treatment/rehabilitation plans (70, 71, 74, 75, 77), questionnaires (24, 68, 69, 76), and interviews (72, 73).

Several studies reported limited agreement between the goals that participants identified, and goals developed by practitioners (24, 69, 73, 74, 76), with the highest levels of agreement for goals addressing symptoms and the lowest level of agreement for religious or spiritual goals (72). One large study (68) reported that nearly half of the inpatient participants had competitive employment goals, with only a third of practitioners agreeing these goals were realistic; practitioners identified more barriers and more significant support needs to be required. In particular, one large qualitative study (75) noted that whilst 70% of written goals included observable behaviors, only 7.8% of these goals contained a measurable criterion. These differences in the individualization of goals were attributed to variances in practices, both between programs and among staff within programs (75). A small qualitative study (70) identified the complexity of creating collaborative treatment plans that recorded individual goals in a way that was actionable, understandable by other practitioners, and met regulatory requirements. As a result, authors highlighted the need for practitioner training (73, 75), with particular attention paid to supporting practitioners to apply motivational approaches to goal and treatment planning (70). A study reporting on the impact of a practitioner training program focused on increasing collaboration in goal planning resulted in a shift in both the content of the goals (increased focus on individual priorities) and the use of first-person language (77), indicating that practitioner training can be effective in improving collaboration and concordance in goal planning.

In all studies, service users were able to identify and set realistic goals. Diagnosis or severity of illness did not appear to impact the ability of participants to plan goals (74). Given these outcomes, authors highlighted difficulties with current service delivery using psychiatric diagnoses to guide the development of interventions (24), recommending that service users’ goals and priorities should guide how services are delivered (69, 72). High levels of service user involvement in goal planning and concordance between participant and practitioner goals resulted in higher levels of goal achievement (69, 74, 75).

## 4 Discussion

This review is, to the best of our knowledge, the first systematic review exploring the use of goal planning in mental health service delivery, and it aimed to provide a better understanding of the types, effectiveness, and experiences of goal planning, as well as the barriers and facilitators to goal planning in mental healthcare. Data from 54 studies were synthesized, demonstrating that goal planning was used in a range of settings, by health practitioners from varied professional backgrounds, and with people experiencing a range of mental illnesses. Overall, findings suggest that there are benefits associated with the use of goal planning in mental healthcare settings.

Interventions that utilized goal planning tended to be complex and varied substantially in approach and application, thus making it challenging to isolate goal planning from the other activities undertaken in mental health service delivery settings. For example, interventions based on psychiatric rehabilitation principles (31–33) provided participants or staff with training to identify goals, describe the skills and resources needed to attain goals, plan, and carry out interventions and access ongoing support to maintain goal attainment. Other studies (66, 67, 70) focused on the impact of the quality of relationships between staff and participants (working alliance), how psychopathology or symptoms effect goal planning and attainment (33, 36, 40, 59–62, 64, 65), or the quality of written goals (42, 77).

The heterogeneity in goal planning processes and measurement of goal attainment precluded direct comparisons across studies or a clear understanding of the impact of goal planning on mental health outcomes. However, studies identified goal planning as an important element in supporting change for people experiencing mental illness, providing a structure that encouraged change and supported motivation to achieve outcomes (28) and improved quality of life (27, 67).

This review highlighted the broad range of goals identified and prioritized by service users and recognized that there was often a divergence between the priorities and goals planned by service users and health practitioners. Goals designed by health practitioners were often focused on symptom management whilst service users prioritized goals for other areas of their everyday lives, including employment, education, relationships, physical health, and living skills, in addition to symptom relief and medication use (45, 49). Service users also valued goals that were individualized to their particular needs, consistent with principles of recovery and recovery-oriented care. Improving goal alignment through shared decision making and collaboration in goal planning promoted increased engagement and retention with services, improved adherence to treatments, increased satisfaction, and enhanced outcomes for service users (49, 53, 57). Factors related to recovery such as focusing on strengths and positive outcomes (29, 40), individualized and personal goals (27, 41–45, 50, 53, 55), collaborative relationships and goal planning (31, 35, 42, 43, 66, 67), informed choice (26), recovery language (77), and partnerships with service users (51) were highlighted across the studies. Whilst some interventions focused on increasing aspects of these in goal planning processes, further research is needed to understand how best to incorporate individual goals into recovery-oriented interventions and service delivery, particularly how best to measure effectiveness and outcomes. The disparity in practitioner and personal understandings of goals and priorities is an important issue that needs review across all levels of service delivery to better align treatment with service user needs (24, 54). This may lead to the development of new and more acceptable treatment options for people experiencing mental illnesses.

Creating collaborative treatment plans that could be individually recorded and understandable by service users and other health practitioners was identified as a complex process (70), one that may have intrinsic therapeutic value in itself (27). As a result, the need for practitioner training was highlighted (73, 75), with particular attention paid to supporting practitioners to develop strong working alliances with service users (66, 69) and apply motivational approaches to goal and treatment planning (70). Studies identified that health practitioners who were provided with training demonstrated increased collaboration during goal planning (31, 42, 43, 77) and improved practitioner confidence in goal planning processes (33). Additionally, due to the individual and diverse goals prioritized by service users, it was identified that practitioners required skills in communicating and coordinating care to ensure that service users were able to access support relevant to their goal outcomes (51, 52, 54). Further understanding of appropriate skills and training needed to support collaborative and effective goal planning is needed.

A number of common aspects of goal planning were identified through this review that mapped easily to the stages reported in the Goal Setting and Action Planning Framework (78). Whilst this practice framework was developed to support the achievement of rehabilitation goals (78), these findings support the applicability of this framework to mental healthcare settings. Stage 1 (goal negotiation) was supported by the provision of appropriate information to allow informed decision-making by service users (26, 30, 34), service user involvement and collaboration in goal planning (67, 69, 74, 75), and shared decision making approaches (49, 53, 57). Stage 2 (goal setting) required an individualized approach (27, 41, 43, 44) that identified the priorities and personal motivations of service users (45, 50, 53, 55, 64, 65) and resulted in the planning of specific goals (28, 59, 61). Stage 3 (planning and action) needed the identification of specific steps that broke complex goals into smaller, achievable steps that enabled achievement (25, 28, 63), focused on positive outcomes rather than obstacles (29, 40), and was supported by a strong working alliance (66), that utilized motivational support approaches (70). Stage 4 (appraisal and feedback) required ongoing evaluation of goal progress and adjustment of goals as needed (30).

### 4.2 Strengths and limitations

The findings from this review need to be considered within the context of its strengths and limitations. This review followed the process outlined in a peer-reviewed protocol (18) and adhered to the PRISMA guidelines (16). Additionally, quality checking of screening and data extraction along with a quality assessment of the studies, added transparency and rigor to the research. The synthesis of data concentrated on both the effects and experiences of goal planning, examining these aspects regarding the use of goal planning in mental healthcare. This focus has enabled recommendations that are specific to the use of goal planning in mental healthcare.

Both a strength and limitation of this review were that evidence was systematically collected from a range of studies, including those with quantitative, qualitative, and mixed method designs. The breadth of studies provided an enhanced understanding of the different contexts in which goal planning is utilized as well as providing insights into the experiences of goal planning from practitioner and service user perspectives. Whilst the inclusion of a diverse range of studies added depth to our understanding; the authors acknowledge the inherent weaknesses of mixed methods systematic reviews (79). Only studies published in English were included, which may have resulted in the loss of important information in different cultural contexts. During synthesis, the study’s primary focus was used to determine the most appropriate theme category for each study. Whilst this allowed for better management of data, the authors recognize the limiting data extraction impacts of this process including the possibility of bias in coding decisions made by researchers and loss of information that could have added to other themes. Given the range of diagnoses and health practitioners within the included studies, the transferability of recommendations is limited. Additionally, a major limitation was a lack of comparability between studies due to heterogeneity of research aims and methods, resulting in difficulty in confidently identifying factors that contributed to effective outcomes. Further research comparing different goal planning interventions is needed to understand which approaches are most effective.

## 5 Conclusion

In conclusion, our systematic integrative review examined the use of goal planning in mental healthcare and found some support for the use of goal planning to improve outcomes in mental healthcare. Individualized, recovery-oriented and collaborative goal planning has been identified as best practice but does not always occur. Goal planning can be a complex process, and mental health practitioners require a range of skills to collaborate and appropriately identify and support service user goal priorities. Additionally, there does not appear to be one standardized approach to goal planning in mental healthcare. Therefore, more research is required to clarify best practice methods for goal planning and required professional education to implement appropriate, recovery-oriented goal planning.

## Data availability statement

The original contributions presented in this study are included in the article/Supplementary material, further inquiries can be directed to the corresponding author.

## Author contributions

VS: study design, title and abstract screen, full-text screen, quality appraisal, data extraction and analysis, and write manuscript. SM: study design, quality appraisal, data analysis, and write manuscript. JH: quality appraisal and comment on manuscript. RN: title and abstract screen, and comment on manuscript. SE-D and CO’R: study design, comment, and edit manuscript. AW: study design, quality appraisal, and write manuscript. All authors contributed to the article and approved the submitted version.
