# Supplementary material for: Goal planning in mental health service delivery: A systematic integrative review
Source: Front Psychiatry. 2022 Dec 19;13:1057915. doi: 10.3389/fpsyt.2022.1057915 (PMC9807176; doi:10.3389/fpsyt.2022.1057915)
Supplement: Supplementary file 2 [file Table_2.DOC]

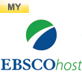
12/03/2022 Print Search History: EBSCOhost

Thursday, March 12, 2022 9:38:53 PM

**# Query Limiters/Expanders Last Run Via Results**

S13 S6 AND S12 Expanders - Apply Interface - EBSCOhost 244

equivalent subjects Research Databases Search modes - Search Screen - Advanced Boolean/Phrase Search

Database - CINAHL Complete

S12 S10 AND S11 Expanders - Apply Interface - EBSCOhost 13,939

equivalent subjects Research Databases Search modes - Search Screen - Advanced Boolean/Phrase Search

Database - CINAHL Complete

S11 set* OR plan* OR attain* Expanders - Apply Interface - EBSCOhost 1,921,644

OR achieve* OR assess* equivalent subjects Research Databases

OR orient* OR commit* Search modes - Search Screen - Advanced OR “action plan” OR Boolean/Phrase Search

“recovery plan” Database - CINAHL Complete

S10 S7 OR S8 OR S9 Expanders - Apply Interface - EBSCOhost 21,571

equivalent subjects Research Databases Search modes - Search Screen - Advanced Boolean/Phrase Search

Database - CINAHL Complete

S9 (MH "Decision Making, Expanders - Apply Interface - EBSCOhost 1,995

Shared") equivalent subjects Research Databases

Search modes - Search Screen - Advanced Boolean/Phrase Search

Database - CINAHL Complete

S8 (MH "Patient Care Plans") Expanders - Apply Interface - EBSCOhost 7,135

equivalent subjects Research Databases Search modes - Search Screen - Advanced Boolean/Phrase Search

Database - CINAHL Complete

S7 (MH "Goals and Expanders - Apply Interface - EBSCOhost 12,606

Objectives") equivalent subjects Research Databases

Search modes - Search Screen - Advanced Boolean/Phrase Search

Database - CINAHL Complete

12/03/2022 Print Search History: EBSCOhost

S6 ( “mental illness*” OR Expanders - Apply Interface - EBSCOhost 57,582

“mental health condition*” equivalent subjects Research Databases

OR “mental health Search modes - Search Screen - Advanced diagnosis” OR “psychiatric Boolean/Phrase Search

illness*” OR “psychiatric Database - CINAHL Complete disorder*” OR “psychiatric

condition*” OR “psychiatric diagnosis” OR “mental health consumer*” OR “mental health service user*” ) NOT ( child* OR youth* OR adolescent* )

S5 “mental illness*” OR Expanders - Apply Interface - EBSCOhost 71,708

“mental health condition*” equivalent subjects Research Databases

OR “mental health Search modes - Search Screen - Advanced diagnosis” OR “psychiatric Boolean/Phrase Search

illness*” OR “psychiatric Database - CINAHL Complete disorder*” OR “psychiatric

condition*” OR “psychiatric diagnosis” OR “mental health consumer*” OR “mental health service user*”

S4 s1 NOT ( child* OR youth* Expanders - Apply Interface - EBSCOhost 48,297

OR adolescent* ) equivalent subjects Research Databases

Search modes - Search Screen - Advanced Boolean/Phrase Search

Database - CINAHL Complete

S3 (MH "Mental Health") Expanders - Apply Interface - EBSCOhost 31,317

NOT ( child* OR youth* equivalent subjects Research Databases

OR adolescent* ) Search modes - Search Screen - Advanced

Boolean/Phrase Search

Database - CINAHL Complete

S2 (MH "Mental Health") Expanders - Apply Interface - EBSCOhost 41,474

equivalent subjects Research Databases Search modes - Search Screen - Advanced Boolean/Phrase Search

Database - CINAHL Complete

S1 (MH "Mental Disorders") Expanders - Apply Interface - EBSCOhost 60,739

equivalent subjects Research Databases Search modes - Search Screen - Advanced Boolean/Phrase Search

Database - CINAHL Complete

25.03.22 Scopus search

( ( ( ( TITLE-ABS-KEY ( "mental illness*" )  AND NOT  TITLE-ABS-KEY ( child*  OR  youth*  OR  adolescent* ) ) )  OR  ( ( TITLE-ABS-KEY ( "mental health*" )  AND NOT  TITLE-ABS-KEY ( child*  OR  youth*  OR  adolescent* ) ) )  OR  ( ( TITLE-ABS-KEY ( "mental disorder*" )  AND NOT  TITLE-ABS-KEY ( child*  OR  youth*  OR  adolescent* ) ) ) )  OR  ( ( ( TITLE-ABS-KEY ( "mental health condition*" )  AND NOT  TITLE-ABS-KEY ( child*  OR  youth*  OR  adolescent* ) ) )  OR  ( ( TITLE-ABS-KEY ( "mental health diagnosis" )  AND NOT  TITLE-ABS-KEY ( child*  OR  youth*  OR  adolescent* ) ) )  OR  ( ( TITLE-ABS-KEY ( "psychiatric diagnosis*" )  AND NOT  TITLE-ABS-KEY ( child*  OR  youth*  OR  adolescent* ) ) ) )  OR  ( ( ( TITLE-ABS-KEY ( "psychiatric illness*" )  AND NOT  TITLE-ABS-KEY ( child*  OR  youth*  OR  adolescent* ) ) )  OR  ( ( TITLE-ABS-KEY ( "psychiatric disorder*" )  AND NOT  TITLE-ABS-KEY ( child*  OR  youth*  OR  adolescent* ) ) )  OR  ( ( TITLE-ABS-KEY ( "psychiatric condition*" )  AND NOT  TITLE-ABS-KEY ( child*  OR  youth*  OR  adolescent* ) ) ) )  OR  ( ( ( TITLE-ABS-KEY ( "psychiatric diagnosis*" )  AND NOT  TITLE-ABS-KEY ( child*  OR  youth*  OR  adolescent* ) ) )  OR  ( ( TITLE-ABS-KEY ( "mental health consumer*" )  AND NOT  TITLE-ABS-KEY ( child*  OR  youth*  OR  adolescent* ) ) )  OR  ( ( TITLE-ABS-KEY ( "mental health service user*" )  AND NOT  TITLE-ABS-KEY ( child*  OR  youth*  OR  adolescent* ) ) ) ) )  AND  ( ( TITLE-ABS-KEY ( set*  OR  plan*  OR  attain*  OR  achieve*  OR  assess*  OR  orient*  OR  commit*  OR  “action  AND plan”  OR  “recovery  AND plan” ) )  AND  ( TITLE-ABS-KEY ( goal*  OR  "care plan*"  OR  "shared decision mak*" ) ) )

Embase Session Results

**No.**

**Query**

**Results**

**2,229**

**#6**

**#4** AND **#5**

**20,873**

**#5**

(**'goal attainment'**/exp OR **'patient care planning'**/exp OR **'shared decision making'**/exp) AND (**set***:ti,ab,kw,jt OR **plan***:ti,ab,kw,jt OR **attain***:ti,ab,kw,jt OR **achieve***:ti,ab,kw,jt OR **assess***:ti,ab,kw,jt OR **orient***:ti,ab,kw,jt OR **commit***:ti,ab,kw,jt OR **'action plan'**:ti,ab,kw,jt OR **'recovery plan'**:ti,ab,kw,jt)

**2,273,878**

**#4**

**#1** OR **#2** OR **#3**

**81,301**

**#3**

((**'mental disease'**/exp OR **'mental health'**/exp OR **2,162,814**) AND **15** AND **mar** AND **2021** AND **'mental illness*'**:ti,ab,kw OR **'mental health condition*'**:ti,ab,kw OR **'mental health diagnosis'**:ti,ab,kw OR **'psychiatric illness*'**:ti,ab,kw OR **'psychiatric disorder*'**:ti,ab,kw OR **'psychiatric condition*'**:ti,ab,kw OR **'psychiatric diagnosis'**:ti,ab,kw OR **'mental health consumer*'**:ti,ab,kw OR **'mental health service user*'**:ti,ab,kw) NOT (**child***:ti,ab,kw,jt OR **youth***:ti,ab,kw,jt OR **adolescent***:ti,ab,kw,jt)

**157,219**

**#2**

**'mental health'**/exp NOT (**child***:ti,ab,kw,jt OR **youth***:ti,ab,kw,jt OR **adolescent***:ti,ab,kw,jt)

**2,184,860**

**#1**

**'mental disorder'**/exp NOT (**child***:ti,ab,kw,jt OR **youth***:ti,ab,kw,jt OR **adolescent***:ti,ab,kw,jt)
